# Integrative Analysis of ^18^F-FDG PET Radiomics and mRNA Expression in Recurrent/Metastatic Oral Squamous Cell Carcinoma: A Cross-Sectional Study

**DOI:** 10.1007/s11307-025-02012-5

**Published:** 2025-05-14

**Authors:** Mai Kim, Wenchao Gu, Reika Kawabata- Iwakawa, Shinichiro Kina, Takahito Nakajima, Tetsuya Higuchi, Masaru Ogawa, Keisuke Suzuki, Yoshito Tsushima, Satoshi Yokoo

**Affiliations:** 1 Department of Oral and Maxillofacial Surgery, and Plastic Surgery, Gunma University Graduate School of Medicine, Maebashi, Japan; 2 Department of Artificial Intelligence Medicine, Graduate School of Medicine, Chiba University, Chiba, Japan; 3 Department of Diagnostic and Interventional Radiology, University of Tsukuba, Tsukuba, Ibaraki Japan; 4 Division of Integrated Oncology Research, Gunma University Initiative for Advanced Research (GIAR), Gunma University, Maebashi, Japan; 5 Department of Diagnostic Radiology and Nuclear Medicine, Gunma University Graduate School of Medicine, Maebashi, Japan; 6 Department of Pharmacology, Graduate School of Medicine, University of the Ryukyus, Okinawa, Japan

**Keywords:** ^18^F-FDG PET, Texture analysis, Oral squamous cell carcinoma, Next-generation sequencing

## Abstract

**Background:**

This study explored the relationship between mRNA expression profiles obtained through next-generation sequencing (NGS) and ^18^F-fluorodeoxyglucose positron emission tomography (^18^F-FDG PET) texture analysis in patients with treatment-resistant oral squamous cell carcinoma (OSCC) who were treated with molecular-targeted drugs. We analyzed the correlation between ^18^F-FDG PET texture features and NGS data in a small cohort of five patients with recurrent or metastatic OSCC who received molecular-targeted drugs after surgery. Patients were categorized into two groups based on treatment response: responders (*n = *3) and non-responders (*n = *2). To validate our findings, we examined transcriptomic data from 292 OSCC patients in The Cancer Genome Atlas (TCGA) database.

**Results:**

The gene ankyrin repeat and SOCS box containing two (ASB2) was significantly overexpressed in non-responders and strongly correlated with specific PET radiomic features, including GLRLM_GLNU, GLRLM_RLNU, and GLZLM_GLNU (*p < *0.05). High ASB2 expression was also associated with poor prognosis in OSCC patients (*p < *0.05) and decreased overall survival, as shown by Kaplan–Meier analysis of the TCGA database (*p = *0.017).

**Conclusions:**

Integrating ASB2 expression data with ^18^F-FDG PET texture features could potentially improve the prediction of treatment outcomes in treatment-resistant OSCC patients undergoing molecular-targeted therapy.

**Supplementary Information:**

The online version contains supplementary material available at 10.1007/s11307-025-02012-5.

## Background

Surgical resection is the standard treatment for oral squamous cell carcinoma (OSCC). However, chemoradiation is preferred for locally advanced, unresectable, recurrent, or metastatic OSCC. Chemotherapeutic agents include cell-killing anticancer agents, molecularly targeted agents, and immune checkpoint inhibitors, depending on the duration of treatment and Combined Positive Score (CPS) [1]. Therapy efficacy is typically evaluated through imaging based on the Response Evaluation Criteria in Solid Tumors (RECIST), with adjustment to second-line treatments if adverse events or disease progression occurs [2]. The primary goals for managing treatment-resistant OSCC are disease control and improved quality of life.

In cases of treatment-resistant OSCC, oncogene panel testing using next-generation sequencing (NGS) with formalin-fixed paraffin-embedded specimens is clinically employed to guide drug therapy selection, with tumor gene mutation burden (TMB) serving as a key indicator [3, 4]. Molecular profiling of therapeutic target genes and genomic mutations in metastatic cancers is beginning to reveal the genetic abnormalities specific to head and neck cancers compared to other malignancies [5, 6]. Additionally, expression profile analysis has been used to compare differentially expressed genes (DEGs) in OSCC patients versus normal controls has been conducted using the large database of The Cancer Genome Atlas (TCGA) [7]. Our previous work explored ferroptosis in OSCC and validated independent prognostic factors in multidisciplinary cancer treatment using the TCGA database [8].

Noninvasive ^18^F-fluorodeoxyglucose positron emission tomography (^18^F-FDG PET) imaging is a valuable biomarker for detecting recurrence and metastasis after head and neck cancer treatment, offering insights into staging, overall survival, and disease-free survival (DFS) [9–11]. Several studies have focused on evaluating residual mass viability through ^18^F-FDG PET functional metabolic imaging [12, 13]. There is increasing interest in monitoring cancer therapy through early noninvasive functional metabolic imaging in treatment-resistant OSCC. Recent reports have explored the relationship between comprehensive genomic and genetic analyses and ^18^F-FDG PET texture analysis in solid tumors [14, 15]. The combined utility and clinical significance of radiogenomics, tumor heterogeneity, and ^18^F-FDG PET metabolic imaging are under investigation [16, 17]. However, the effectiveness of optimizing treatment algorithms through radiogenomics, integrating FDG PET texture analysis and NGS in treatment-resistant OSCC, and the role of metabolic imaging in determining treatment efficacy have not been fully explored.

This study aims to investigate the relationship between mRNA expression using NGS and ^18^F-FDG PET texture analysis in patients with treatment-resistant OSCC receiving molecular-targeted drugs.

## Methods

### Patient Characteristics

**Fig. 1:**
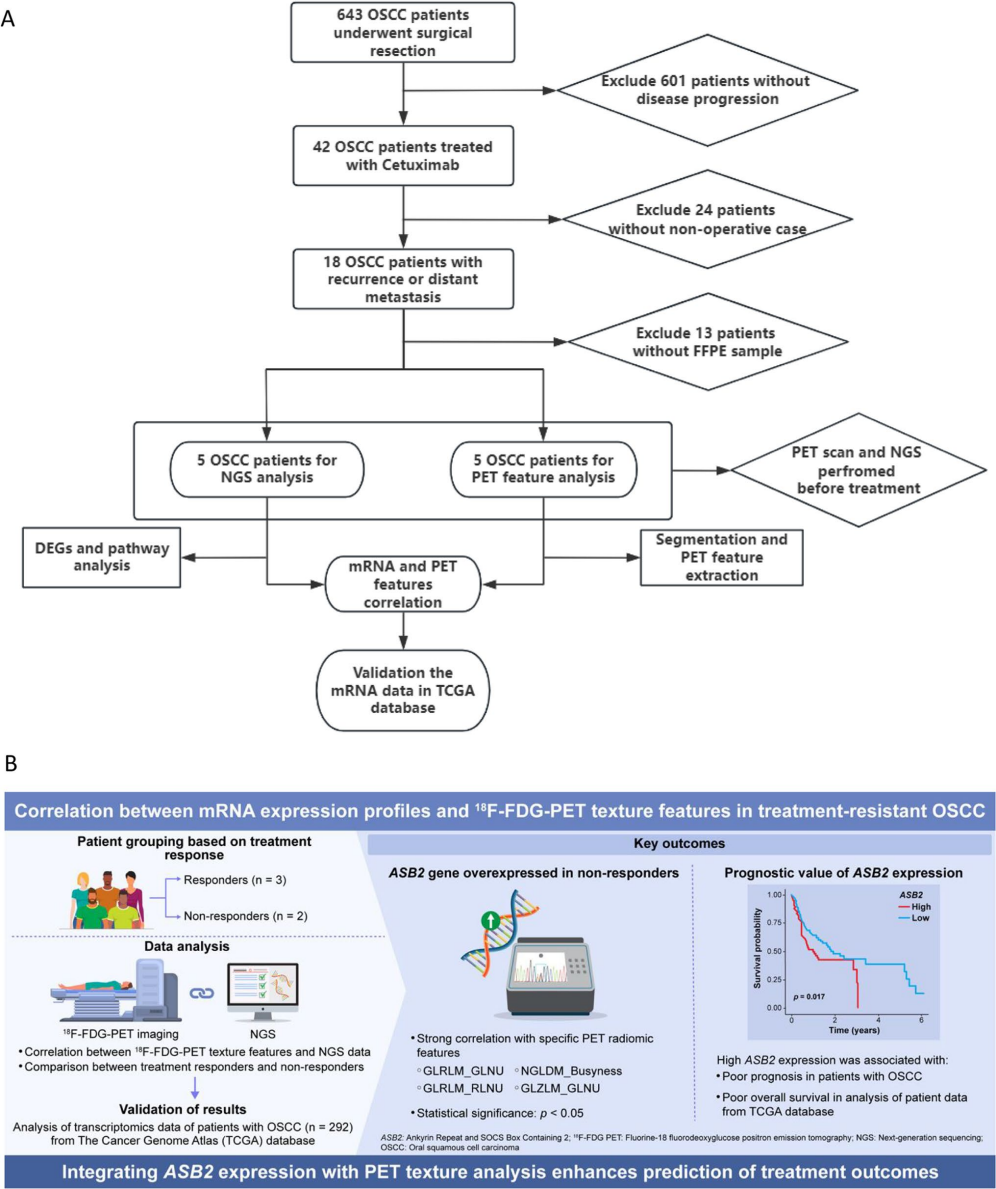
**A**. Flow diagram of patient enrollment process. Detailly, five patient were selected from our 643 patients database. All the PET imaging and NGS samples were acquired and analysis before treatment. **B**. Graphic abstract

Among the 643 patients who underwent initial surgical treatment for OSCC at our institute between January 2010 and January 2020, 42 patients required treatment due to disease progression following initial therapy between April 2015 and January 2020. Of these, 18 patients developed postoperative recurrence or distant metastasis and were subsequently treated with cetuximab. This study specifically focuses on a subset of five patients who underwent ^18^F-FDG PET/CT imaging prior to initiating treatment for recurrence or metastasis and for whom comprehensive NGS analysis data were available (Fig. 1 A-B).

Study subjects were categorized into two groups based on the RECIST diagnostic criteria: response group (*n = *3, Complete Response [CR] and Partial Response [PR]) and non-response group (*n = *2, Stable Disease [SD] and Progressive Disease [PD]). NGS was performed on formalin-fixed specimens, and ^18^F-FDG PET texture features (66 parameters) were analyzed using LifeX (version 7. 6. 0). For validation, 292 OSCC cases were included from 527 head and neck cancer cases with genetic analysis available in The Cancer Genome Atlas (TCGA).

### ^18^F-FDG PET Imaging Acquisition

^18^F-FDG PET imaging was performed using Biograph 64 (Siemens Healthcare, Germany) or Milkyway (GE, Germany). All patients fasted for 6 h before imaging and then received intravenous ^18^F-FDG at 4.0 MBq/kg, followed by a 60-min resting period before imaging. Image analysis was conducted by a certified PET nuclear medicine physician experienced in head and neck imaging, utilizing the Syngo via Multimodality Workplace analysis software (Siemens Healthcare GmbH, Erlangen, Germany).

### Segmentation and Texture Extraction

Segmentation and feature extraction methods were similar to those previously reported [18]. The DICOM imaging was retrieved from the PACS system in our institution. The volume of interest (VOI) was delineated manually by an investigator with over 10 years of experience PET imaging analysis, and feature extraction was then performed automatically using LIFEx software (https://www.lifexsoft.org/index.php) with the following parameters: 64 grey levels, bin size of 0.3125, and intensity rescaling with a minimum SUV of 0 and a maximum SUV of 40. The region of interest for the lesion was measured using the volume of interest and was set above a threshold of 40% SUVmax. The resampling size was 4.7 × 4.7 × 3.3 mm. Radiomics features were then extracted. Details of the radiomic feature descriptions are provided in Supplementary Table 1.

### RNA Isolation and RNA-seq Analysis

Total RNA was extracted from macroscopically dissected formalin-fixed paraffin-embedded (FFPE) tissue samples using the High Pure FFPET RNA Isolation Kit (Roche, Basel, Switzerland) following the manufacturer's protocol. RNA quality was assessed using the Agilent RNA 6000 Pico Kit (Agilent Technologies) and the Agilent Bioanalyzer (Agilent Technologies), confirming that the RNA was of sufficient quality for RNA-seq analysis.

RNA (150 ng) was processed to create sequencing libraries with barcoded fragments using the QIAseq FastSelect-rRNA HMR Kit (QIAGEN) and KAPA RNA HyperPrep Kit (KAPA Biosystems), following the manufacturer's guidelines. Paired-end 75 bp reads were generated on a NextSeq 500 System (Illumina) with the NextSeq 500 High Output v2.5 Kit (Illumina). FastQC (v0.11.1) was used to assess sequence quality prior to alignment. The reads were aligned to the UCSC reference human genome 19 (hg19) with STAR (v2.5.3a, DNASTAR, Inc.) and quantified with RSEM (v1.3.3). Both samples had over 73 million input reads.

Count data were transformed into transcripts per million (TPM) based on previously described methods. Differential expression analysis was performed using the “Dseq2” R package with a threshold of adjusted *p*-value < 0.05 (Bonferroni correction) and log fold change > 1.5. KEGG (Kyoto Encyclopedia of Genes and Genomes) and Gene set enrichment analysis (GSEA) were conducted to investigate biological differences between response and non-response groups. Shapiro–Wilk normality test was performed to confirm the normality of radiomics features and gene expression. Spearman correlation analysis was used to determine the relationship between gene expression and radiomic features. Radiomics-related genes associated with survival in OSCC patients were confirmed using TCGA-OSCC mRNA data (*n = *292) obtained from the TCGA-HNSCC dataset (GDC, http://portal.gdc.cancer.gov/), based on the previously described tumor location in the oral cavity [8]. The details of patient’s characteristics could be found in Supplementary Table 2.

### Statistical Analysis

All analyses were performed using R software (Version 4.3.1). Spearman correlation test was compared between the two groups. Two groups comparison was used the Mann–Whitney U test. Survival analysis was conducted using the Kaplan–Meier method, and significance of differences was evaluated using the log-rank test. The heatmap was generated using the “pheatmap” package. Bonferroni correction was applied, and a *p*-value adjusted to < 0.05 was considered statistically significant.

## Results

**Fig. 2:**
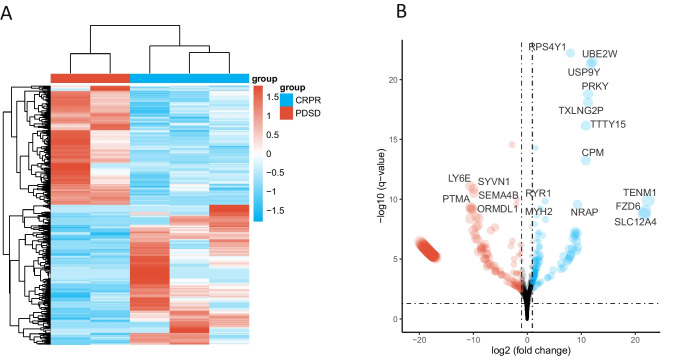
Differentially expressed genes (DEGs) in responder and non-responder groups. The ratio of DEG expression levels is plotted on the x-axis, while statistical significance is shown on the y-axis. **A**. Heatmap illustrating the expression levels of significantly differentially expressed genes (adjusted *p*-value < 0.05) in responder and non-responder groups. Rows represent DEGs, and columns represent individual patients, with hierarchical clustering applied to both rows and columns to reveal expression patterns. The color scale indicates relative expression levels. **B**. Volcano plot showing the distribution of DEGs between responder and non-responder groups. Genes with significant differential expression (adjusted *p*-value < 0.05 and log2 fold-change > 1.5) are highlighted, with upregulated genes in the responder group shown in red and downregulated genes in blue. Non-significant genes are shown in black

Of the five eligible patients with available NGS analysis, three had primary lesions were on the tongue, and one had a lesion on the buccal mucosa, and floor of mouth. All lesions were classified as highly differentiated OSCC (Table 1). Among these patients, there were three responders (complete response [CR]: 1, partial response [PR]: 2) and two non-responders (stable disease [SD]: 2). All patients received treatment with cetuximab. We identified 952 DEGs between the responder and non-responder groups (Fig. 2A).

**Table 1:** Patient characteristics

Variables	All patients
	(*n = *5)
Age (median, quartile), years	76.0(61.0 ± 82.5)
Gender, n	
Male	4
Female	1
Site of primary tumor	
Tongue	3
Floor of mouth	1
Buccal mucosa	1
Stage*	
I/II/III/IV	1/1/3/0
Overall response	
CR/PR/SD/PD	1/2/2/0
Term during from surgery to metastasis (median, quartile), days	324.0 (305.0 ± 459.0)
Number of times cetuximab was administered (median, quartile), times	25.0(24.0 ± 49.0)

^*^No patients were classified as Stage 0, IV

**Fig. 3:**
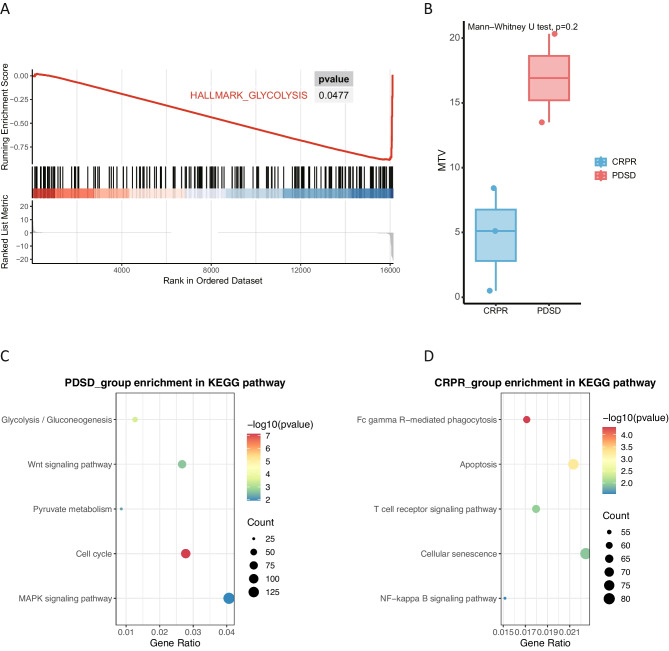
**A-B**. GSEA enrichment of glycolysis pathway and MTV distribution in non-responder and responder groups. Each gene was plotted along the x-axis, with the enrichment score represented on the y-axis. **C-D**. KEGG analysis in non-responder and responder groups.

Volcano plots illustrated the DEGs in both groups (Fig. 2B). Statistically significant differences were observed in the expression of TENM1, FZD6, SLC12 A4, LY6E, and PTMA between the two groups. GSEA was conducted to assess biological functions (Fig. 3A). The non-responder group exhibited glycolytic activation; consistent with ^18^F-FDG PET imaging results. The metabolic tumor volume (MTV) tended to be higher in the non-responder group (Fig. 3B, *p* = 0.2). Moreover, the KEGG analysis results show that non-responder group enriched in cell proliferation (Cell cycle), cancer pathway (MAPK and WNT signaling pathway), cell metabolism (Pyruvate metabolism). In contrast, the responder group show that most activated pathway in immune response (Fc gamma R-mediated phagocytosis, T cell receptor and NF-kB signaling pathway) and cell apoptosis (Fig. 3 C-D).

**Fig. 4:**
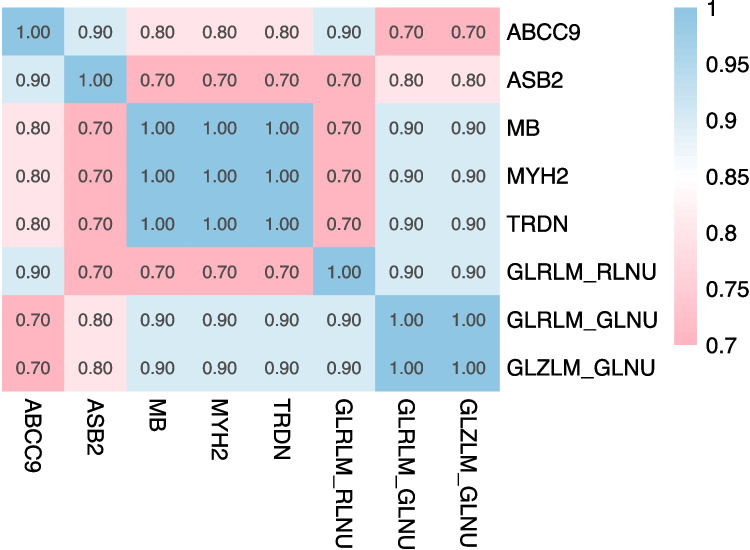
Correlation of ^18^F-FDG PET features with DEGs using Spearman correlation analysis. The figure illustrates the relationship between quantitative PET imaging features and the expression levels of DEGs identified in the study. Each number represents a specific gene-feature pair, with the strength and direction of the correlation denoted by the Spearman correlation coefficient (r).The analysis provides insights into the potential association between PET imaging biomarkers and gene expression profiles

**Fig. 5:**
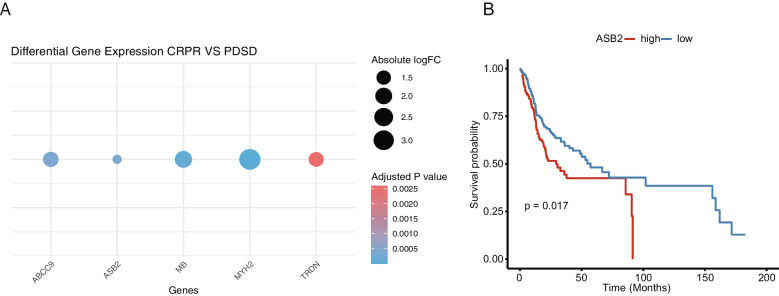
ASB2 Expression and Survival Analysis in Responder Group and OSCC Patients. **A**. Dotplot showing significantly lower ASB2, TRDN, MYH2, MB, and ABCC9 expression in the responder group, which includes five patients enrolled at our institution. **B**. Kaplan–Meier analysis of overall survival (OS) in patient groups with high and low ASB2 expression levels, based on a cohort of 292 patients with oral squamous cell carcinoma (OSCC)from the TCGA database

We further investigated the radiomic features associated with DEGs using Spearman correlation analysis, with a threshold of adjust-*p < *0.05 and r > 0.6. Significant correlation was found between TRDN, MYH2, MB, ABCC9 and ASB2 with GLRLM_GLNU, GLRLM_RLNU and GLZLM_GLNU (Fig. 4). Additionally, all five genes expression was higher in the non-responder group compared to the responder group (Fig. 5A; adjust_*p < *0.05). Survival analysis of the five genes in TCGA-OSCC cohort showed that high ASB2 expression was associated with significantly worse prognosis (Kaplan–Meier analysis; Fig. 5B; *p = *0.017). In contrast, except for ASB2, the other four genes did not show significant prognostic value (Supplementary Fig. 1; *p* > 0.05).

## Discussion

We investigated the relationship between mRNA expression using NGS and ^18^F-FDG PET texture analysis in treatment-resistant OSCC patients undergoing molecularly targeted therapy. Although MTV has been established as a prognostic factor in previous studies, our analysis may provide supplementary insights to conventional metrics. Specifically, our findings suggest that advanced radiomic features such as GLRLM_GLNU, GLRLM_RLNU, and GLZLM_GLNU may serve useful imaging biomarkers for assessing treatment efficacy. Additionally, the correlation between PET and NGS data with high ASB2 expression indicates the potential to predict poor prognosis. Integrating mRNA data with ^18^F-FDG PET texture features may enhance predictions of OSCC treatment efficacy. Combining these features with extensive databases could enable the creation of subgroups that account for genes expressed related to poor prognostic factors and individual patient risk, proving beneficial for non-invasive applications.

Currently, ^18^F-FDG PET imaging is primarily used for detecting metastases in lymph nodes and distant organs before initial treatment and for assessing regions of interest using SUVmax in OSCC management. Besides morphological imaging modalities such as CT and MRI, integrating functional metabolic changes that reflect tumor glucose metabolism and comprehensive tumor gene analysis through ^18^F-FDG PET may prove valuable for monitoring treatment efficacy. This method is a useful tool for assessing treatment effectiveness. Our study demonstrated the utility of ^18^F-FDG PET texture analysis in correlating features with mRNA expression and confirmed the effectiveness of radiogenomics in treatment-resistant OSCC. The novelty of our study lies in its focus on combining PET texture features with mRNA fusion data to investigate radiogenomic associations in oral squamous cell carcinoma that has progressed despite standard treatment. By integrating these data, we identified potentially useful genes and validated their prognostic significance using external datasets from TCGA. This distinct approach highlights the potential of radiogenomics as a valuable method for understanding tumor biology and evaluating prognosis in challenging clinical scenarios. Additionally, ASB2, a gene strongly correlated with radiomic features, was associated with poor prognosis, corroborated by a large dataset from TCGA.

Jiaving et al. (2021) conducted a survival curve analysis of OSCC patients from The Cancer Genome Atlas (TCGA), identifying six candidate genes (CXCL10, OAS2, IFIT1, CCL5, LRRK2, and PLAUR) associated with patient survival [19, 20]. In this study, NGS analysis of cases treated with molecular-targeted drugs led to the identification of new genes through enrichment analysis. We found that the non-response group exhibited enhanced cancer proliferation and metabolic pathway activity, whereas the response group demonstrated greater immune activation. The diagnostic and prognostic value of mRNA in OSCC, along with its correlation with the tumor's clinicopathological profile, has been documented. Future research should explore these genes’ role in survival and treatment response prediction in OSCC patients. Aberrantly expressed mRNAs influence tumorigenesis, and the PI3 K/Akt signaling pathways and target genes for the repression of the p53 signaling pathway have been identified. Further investigation is necessary to understand therapeutic sensitivity and these signaling pathways.

Radiogenomic features based on SUVmax in FDG PET and the epithelial-mesenchymal transition (EMT) phenotype based on mRNA expression have been studied in non-small cell lung cancer (NSCLC). This study explored the association between radiogenomic features based on SUVmax in ^18^F-FDG PET and EMT based on mRNA expression, confirming that higher EMT protein expression was linked to significantly different cell migration, glucose uptake, and hexokinase activity. Additionally, NSCLC cells exhibited increased resistance to chemotherapy in vitro [21]. In our study, ASB2-positive cases were categorized within the tumor cytoplasm of non-responder group cases, though some reports suggest that ASB2 knockdown inhibits the migration of human NK cells [22]. The efficacy of the tumor microenvironment in immunotherapy, particularly concerning NK cells, warrants further investigation. In colorectal cancer, ASB2α has been reported to induce a tumor-promoting immune pathway via Th2 cells and to facilitate tumor progression through the evasion of immune surveillance [23]. In oral squamous cell carcinoma, Th2-related cytokines, such as IL- 4 and IL- 10, have also been reported to show elevated serum levels compared to healthy individuals [24], suggesting that the expression of these cytokines, involved in cancer immune responses, may contribute to their potential role as prognostic biomarkers reflected in PET imaging findings in OSCC [25].

This study conducted mRNA expression analysis and ^18^F-FDG PET texture analysis treatment-resistant OSCC patients, but the sample size was limited to only five patients. This small sample size is a significant limitation, reducing statistical power and limiting the generalizability of the findings. However, we supplemented our analysis with data from 292 OSCC patients from the TCGA database, which helped validate the associations between ASB2 expression and ^18^F-FDG PET texture features observed in our study. The use of TCGA data adds reliability to our conclusions, despite the small initial sample size. However, future studies should consider public datasets that include both PET imaging and NGS data, as the TCGA-OSCC dataset does not contain PET-CT imaging data.

As an approach to the clinical implementation of radiogenomics, a functional metabolic imaging database based on TMB levels derived from cancer-related genes following standard treatment enables non-invasive and dynamic monitoring. Integrating radiogenomic insights into clinical workflows may facilitate personalized treatment planning by identifying patients most likely to benefit from targeted therapies or immunotherapies and detecting early treatment resistance, thereby enabling timely therapeutic modifications. Furthermore, non-invasive metabolic imaging may enhance patient follow-up by providing real-time assessments of tumor evolution. This process, in turn, supports the development of strategic treatment plans that optimize the timing of drug therapy, treatment costs, and toxicity, potentially contributing to improved survival rates. These applications have the potential to improve treatment efficacy, optimize resource utilization, and enhance clinical outcomes.

Nevertheless, clinical and treatment-related differences between the patient data from our study and the TCGA cohort could influence the results. The conclusions drawn from our five cases need further validation in larger independent cohorts to ensure robustness and broader applicability.

Future research should focus on collecting data both before and after initial treatment and analyzing them longitudinally to better understand the mechanisms underlying treatment resistance and to improve the accuracy of treatment efficacy predictions. By predicting tumor growth rate based on gene mutation levels and stratifying TMB-related therapeutic resistance mechanisms, the improved matching accuracy of functional metabolic imaging will enable healthcare providers to anticipate therapeutic resistance before initiating drug therapy and determine the optimal timing for therapy initiation. Additionally, expanding the study to include a larger patient population will strengthen the findings and ensure their applicability across a broader range of clinical settings.

## Conclusions

In this study, we conducted mRNA and PET texture analyses in patients with treatment-resistant OSCC who were treated with molecular-targeted drugs. We identified a correlation between high ASB2 expression and the specific PET features: GLRLM_GLNU, GLRLM_RLNU, and GLZLM_GLNU. These findings highlight the potential of using ASB2 expression and specific PET features as biomarkers for assessing treatment response and guiding therapeutic strategies in OSCC.

## Supplementary Information

Below is the link to the electronic supplementary material.

Supplementary file1 (DOCX 18 KB)

Supplementary file2 (DOCX 17 KB)

Supplementary file3 (DOCX 17 KB)

Supplementary file4 (DOCX 71 KB)

## Data Availability

The datasets used in this study are available from the corresponding author upon reasonable request.

## Abbreviations

18 F-FDG PET: 18F-fluorodeoxyglucose Positron Emission Tomography
ASB2: Ankyrin Repeat and SOCS Box Containing 2
CPS: Combined Positive Score
CR: Complete Response
CT: Computed Tomography
DEG: Differentially Expressed Gene
DFS: Disease-Free Survival
EMT: Epithelial-Mesenchymal Transition
FFPE: Formalin-Fixed Paraffin-Embedded
GSEA: Gene Set Enrichment Analysis
GLRLM_GLNU: Gray Level Run Length Matrix—Gray Level Non-Uniformity
GLRLM_RLNU: Gray Level Run Length Matrix—Run Length Non-Uniformity
GLZLM_GLNU: Gray Level Zone Length Matrix—Gray Level Non-Uniformity
MRI: Magnetic Resonance Imaging
MTV: Metabolic Tumor Volume
NGS: Next-Generation Sequencing
NSCLC: Non-Small Cell Lung Cancer
OSCC: Oral Squamous Cell Carcinoma
PD: Progressive Disease
PET: Positron Emission Tomography
PR: Partial Response
RECIST: Response Evaluation Criteria in Solid Tumors
RNA-seq: RNA Sequencing
SD: Stable Disease
SUVmax: Maximum Standardized Uptake Value
TCGA: The Cancer Genome Atlas
TMB: Tumor Mutation Burden
TPM: Transcripts Per Million
VOI: Volume of Interest
