# Supplementary material for: Integrative Analysis of 18F-FDG PET Radiomics and mRNA Expression in Recurrent/Metastatic Oral Squamous Cell Carcinoma: A Cross-Sectional Study
Source: Mol Imaging Biol. 2025 May 14;27(3):421–30. doi: 10.1007/s11307-025-02012-5 (PMC12162752; doi:10.1007/s11307-025-02012-5)
Supplement: Supplementary file 1 — Supplementary file1 (DOCX 18 KB) [file 11307_2025_2012_MOESM1_ESM.docx]

Supplementary Table 1. The list of radiomics features.

Radiomics parameters

| Classification | Features |
| --- | --- |
| Conventional Indices | SUVmin |
|  | SUVmean |
|  | SUVstd |
|  | SUVmax |
|  | MTV |
|  | TLG |
|  | SUVKurtosis |
|  | SUVExcessKurtosis |
|  | SUVSkewness |
|  | SUVQ1 (Quartiles 1) |
|  | SUVQ2 (Quartiles 2) |
|  | SUVQ3 (Quartiles 3) |
| Discretized Indices |  |
|  | DISCRETIZED TLG |
|  | DISCRETIZED_SUVKurtosis |
|  | DISCRETIZED_SUVExcessKurtosis |
|  | DISCRETIZED_SUVSkewness |
|  | DISCRETIZED_SUVmin |
|  | DISCRETIZED_SUVmean |
|  | DISCRETIZED_SUVstd |
|  | DISCRETIZED_SUVmax |
|  | DISCRETIZED_SUVQ1 |
|  | DISCRETIZED_SUVQ2 |
|  | DISCRETIZED_SUVQ3 |
| First Order Features |  |
| Histogram Based | HISTO_Skewness |
|  | HISTO_ExcessKurtosis |
|  | HISTO_Kurtosis |
|  | HISTO_Entropy_log10 |
|  | HISTO_Entropy_log2 |
|  | HISTO_Energy |
| Shape Based | SHAPE_Sphericity |
|  | SHAPE_Compacity |
|  | SHAPE_Volume (mL) |
|  | SHAPE_Volume (voxels) |
|  | SHAPE_Surface |
| Second Order Features (PET and CT) |  |
| Grey Level Co-occurrence Matrix (GLCM) | GLCM_Homogeneity |
|  | GLCM_Energy |
|  | GLCM_Contrast |
|  | GLCM_Correlation |
|  | GLCM_Entropy_log10 |
|  | GLCM_Entropy_log2 |
|  | GLCM_Dissimilarity |
| Grey-Level Run Length Matrix (GLRLM) | GLRLM_SRE (Short-Run Emphasis) |
|  | GLRLM_LRE (Long-Run Emphasis) |
|  | GLRLM_LGRE (Low Grey-level Run Emphasis) |
|  | GLRLM_HGRE (High Grey-level Run Emphasis) |
|  | GLRLM_SRLGE (Short-Run Low Grey-level Emphasis) |
|  | GLRLM_SRHGE (Short-Run High Grey-level Emphasis) |
|  | GLRLM_LRLGE (Long-Run Low Grey-level Emphasis) |
|  | GLRLM_LRHGE (Long-Run High Grey-level Emphasis) |
|  | GLRLM_GLNU (Grey-Level Non-Uniformity) |
|  | GLRLM_RLNU (Run Length Non-Uniformity) |
|  | GLRLM_RP (Run Percentage) |
| Neighbourhood Grey-Level Difference Matrix (NGLDM) | NGLDM_Coarseness |
|  | NGLDM_Contrast |
|  | NGLDM_Busyness |
| Grey-Level Zone Length Matrix (GLZLM) | GLZLM_SZE (Short-Zone Emphasis) |
|  | GLZLM_LZE (Long-Zone Emphasis) |
|  | GLZLM_LGZE (Low Grey-level Zone Emphasis) |
|  | GLZLM_HGZE (High Grey-level Zone Emphasis) |
|  | GLZLM_SZLGE (Short-Zone Low Grey-level Emphasis) |
|  | GLZLM_SZHGE (Short-Zone High Grey-level Emphasis) |
|  | GLZLM_LZLGE (Long-Zone Low Grey-level Emphasis) |
|  | GLZLM_LZHGE (Long-Zone High Grey-level Emphasis) |
|  | GLZLM_GLNU (Grey-Level Non-Uniformity) |
|  | GLZLM_ZLNU (Zone Length Non-Uniformity) |
|  | GLZLM_ZP (Zone Percentage) |
