# Supplementary material for: Integrative Analysis of 18F-FDG PET Radiomics and mRNA Expression in Recurrent/Metastatic Oral Squamous Cell Carcinoma: A Cross-Sectional Study
Source: Mol Imaging Biol. 2025 May 14;27(3):421–30. doi: 10.1007/s11307-025-02012-5 (PMC12162752; doi:10.1007/s11307-025-02012-5)
Supplement: Supplementary file 2 — Supplementary file2 (DOCX 17 KB) [file 11307_2025_2012_MOESM2_ESM.docx]

**Supplementary Table 2. Patient characteristics (TCGA-OSCC)**

| Variables | All patients |
| --- | --- |
|  | (n = 5) |
| Age (median, quartile),years | 62.1(61.0±17.0) |
| Gender, n |  |
| Male | 194 |
| Female | 98 |
| Site of primary tumor |  |
| Tongue | 117 |
| Floor of mouth | 59 |
| Buccal mucosa  Alveolar Ridge  Hard Palate  Lip  Oral Cavity | 21  18  7  3  67 |
| Stage |  |
| I/II/III/IV/X | 29/92/53/109/9 |
