# Supplementary material for: Integrative Analysis of 18F-FDG PET Radiomics and mRNA Expression in Recurrent/Metastatic Oral Squamous Cell Carcinoma: A Cross-Sectional Study
Source: Mol Imaging Biol. 2025 May 14;27(3):421–30. doi: 10.1007/s11307-025-02012-5 (PMC12162752; doi:10.1007/s11307-025-02012-5)
Supplement: Supplementary file 3 — Supplementary file3 (DOCX 17 KB) [file 11307_2025_2012_MOESM3_ESM.docx]

| Variables | W | P-value |
| --- | --- | --- |
| ASB2 | 0.76793 | 0.12 |
| ABCC9 | 0.86755 | 0.26 |
| MB | 0.84517 | 0.18 |
| MYH2 | 0.77149 | 0.10 |
| TRDN  GLRLM_GLNU  GLRLM_RLNU  NGLDM_Busyness  GLZLM_GLNU | 0.84752  0.75499  0.87697  0.83398  0.85964 | 0.19  0.33  0.30  0.15  0.23 |

**Supplementary Table 3 Shapiro-Wilk normality test**
