# Supplementary material for: Integrative Analysis of 18F-FDG PET Radiomics and mRNA Expression in Recurrent/Metastatic Oral Squamous Cell Carcinoma: A Cross-Sectional Study
Source: Mol Imaging Biol. 2025 May 14;27(3):421–30. doi: 10.1007/s11307-025-02012-5 (PMC12162752; doi:10.1007/s11307-025-02012-5)
Supplement: Supplementary file 4 — Supplementary file4 (DOCX 71 KB) [file 11307_2025_2012_MOESM4_ESM.docx]

Supplementary Figure 1. Kaplan-Meier analyses OS analysis for high and low TRDN, MYH2, MB and ABCC9 expression
